# Effects of disturbance regime on carbohydrate reserves in meadow plants

**DOI:** 10.1093/aobpla/plv123

**Published:** 2015-10-27

**Authors:** Štěpán Janeček, Alena Bartušková, Michael Bartoš, Jan Altman, Francesco de Bello, Jiří Doležal, Vít Latzel, Vojtěch Lanta, Jan Lepš, Jitka Klimešová

**Affiliations:** 1 Institute of Botany, Academy of Sciences of the Czech Republic, Dukelská 135, CZ-379 82 Třeboň, Czech Republic; 2 Department of Ecology, Faculty of Science, Charles University in Prague, Viničná 7, CZ-128 44 Praha 2, Czech Republic; 3 Faculty of Science, University of South Bohemia, Branišovská 31, CZ-370 05 České Budějovice, Czech Republic; 4 Section of Ecology, Department of Biology, University of Turku, FIN-20014 Turku, Finland

**Keywords:** Carbohydrates, management, meadow, storage organs, TNC concentration, TNC pool

## Abstract

Carbohydrate storage enables plants to tolerate both seasonally unfavourable conditions and recover from disturbance. Although short-term changes in storage levels due to disturbance are fairly well known, less is known about long-term changes in storage levels, especially in response to cessation of repeated disturbance. Additionally, whereas it is presumably the total amount (pool) of storage carbohydrate reserves that is of importance, typically carbohydrate concentrations are measured instead, as a proxy. We assessed changes in carbohydrate concentrations and pools in storage organs and changes in above- versus belowground biomass in response to mowing cessation in nine herbs from two meadows (dry and wet) at the (June) peak of vegetation development and the (October) growing season end 1 and 3 years after the change in the disturbance regime. We tested three hypotheses: (1) storage will increase with abandonment of mowing only in the first year after disturbance cessation, but not further increase subsequently, as high storage would hinder competitive ability; (2) storage will increase towards the end of the season in both disturbed and undisturbed plants; and (3) changes in carbohydrate concentrations are accurate predictors of changes in pools. Although species-specific changes in carbohydrate reserves occurred in the wet meadow, more general trends appeared in the dry meadow. There, plants accumulated higher carbohydrate reserves at the end of the season, especially in unmown plots. However, the reserves for plants in both disturbance regimes were the same at the growing season peak (June) in both examined years. The increase in storage of carbohydrates on unmown plots in October was manifested by increases of both storage organ biomass and carbohydrate concentration, whereas in mown plots, it was due only to increased carbohydrate concentration. Although concentrations and pools represent different aspects of plant carbohydrate economy, concentrations will represent short-term responses to changed disturbance regimes.

## Abstract (precis)

Plants in unmown meadows are able to store large amounts of carbohydrates. These stores, however, become depleted during winter and/or spring and thus do not differ from levels in mown plots at the peak of the next growing season. It is clear, moreover, that although carbohydrate concentrations at first reflect the carbohydrate mobilization needed for resprouting in response to plant damage and then the refilling of reserves thereby expended, the total carbohydrate amounts are affected by the growth of storage organs. Although concentrations and total amounts of carbohydrates reflect different aspects of plant carbohydrate storage, their concentration might sufficiently describe short-term effects of disturbance.

## Introduction

Carbohydrate storage is an important strategy allowing plants to tolerate unpredictable or fluctuating environmental conditions. Such reserves can help plants to survive periods when the production of assimilates is limited, for example, because of damage to photosynthetic organs, thus supporting subsequent resprouting. This function of carbohydrate reserves has been manifested not only in many plant communities where plants experience frequent disturbance by fire (Schutz *et al*. 2009), herbivory (Hassan and Krueger 1980) or traditional mowing (Janeček *et al*. 2011) but also in ecosystems where disturbance is much less common (e.g. in tropical forests, Poorter *et al*. 2010). However, carbohydrate storage has its costs, as it is realized at the expense of current growth, and storing plants are, therefore, generally penalized by having lower competitive ability (Chapin *et al*. 1990; Iwasa and Kubo 1997; Kobe 1997).

The response of carbohydrate storage to defoliation occurs over two timescales: immediate (minutes to days) and long-term (weeks to years). The immediate response of carbohydrate reserves to defoliation was studied using ^14^C labelling, which revealed that carbohydrate reserves support both new growth and respiration of remaining plant parts (Schmitt *et al*. 2013). A decrease in carbohydrate reserves in storage organs after defoliation because of allocation to regrowth has been shown in pot experiments (Moran *et al*. 1953; Gallagher *et al*. 1997; Morvan-Bertrand *et al*. 1999; Janeček and Klimešová 2014) and in field studies (Cooper and Watson 1968; Bartoš *et al*. 2011). The long-term response of carbohydrate reserves to defoliation differs from the immediate response in that it includes not only depletion but also some refilling of storage organs (Bartoš *et al*. 2011).

**Figure 1.**
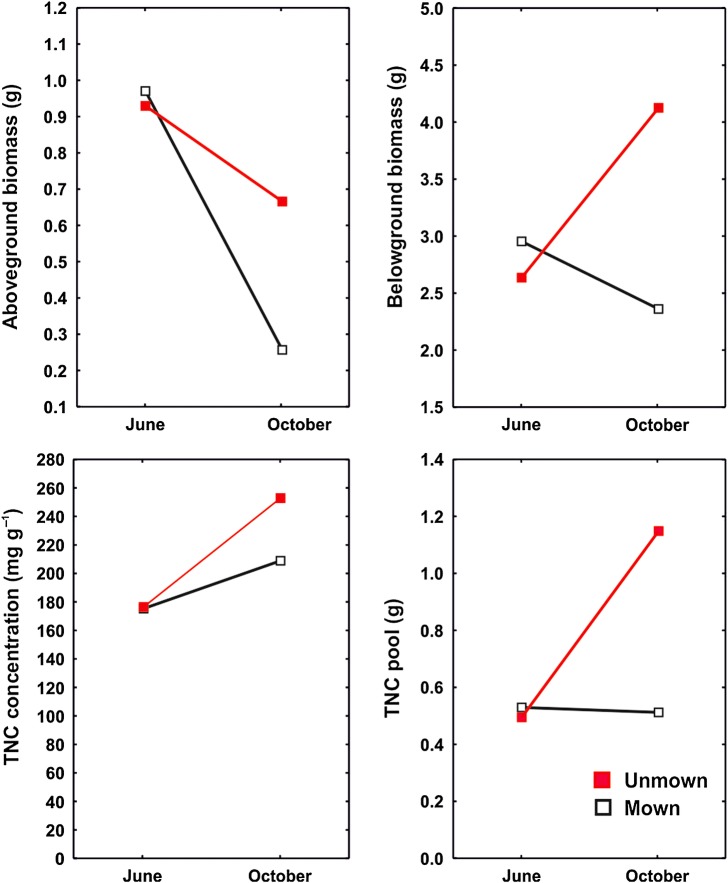
Dry meadow. Effect of treatments on seasonal changes in biomass and carbohydrate reserves.

Along with the important role of carbohydrate reserves in coping with damage, carbon storage is also needed for survival in adverse growing conditions. Plants, therefore, accumulate carbohydrates seasonally before the onset of cold or dry periods to enable respiration as well as prompt regrowth at the end of the adverse period (Mooney and Billings 1960; Janeček *et al*. 2011; Fig. 1. in Latzel *et al*. 2014).

This seasonal aspect of storage raises questions about how it interacts with both short- and long-term responses to damage. Bartoš *et al.* (2011) showed that over a single growing season, the dominant meadow grass accumulated greater storage where mowing disturbance was excluded than where it was mown. However, it is not clear how such a shift in carbohydrate reserves during the growing season will interact with changes in the disturbance regime over the long term.

Iwasa and Kubo (1997), based on their modelling study, suggested that after relaxing disturbance, carbohydrate storage will increase, but not without limits, as the plants' competitive ability will be compromised by the lack of resprouting. However, they did not consider seasonality. Therefore, one of the major issues that we investigate in the present study is the long-term effect of cessation of regular disturbance on ‘seasonal’ carbohydrate reserve levels. However, this question raises a second issue, regarding the assessment of such storage levels. Namely, although it is the overall amount of such reserves that are presumably of biological importance to the plant, typically it is their concentration that is measured, as a proxy. This assessment is performed by taking a sample of the specialized organs, determining the amount of total non-structural carbohydrates (TNCs) found in it, and dividing this by the biomass of the sample. This approach is premised on the fact that carbohydrate reserves are in fact non-structural carbohydrates, and it is employed because it is easier than actually measuring carbohydrate pools (i.e. the actual total weight of non-structural carbohydrates in the storage organs, calculated as TNC concentration multiplied by storage organ biomass).

The relative difficulty of directly measuring storage pools is due to the fact that belowground organs comprise the main storage sites in ecosystems subjected to frequent disturbance removing all aboveground biomass or in which the seasonal resting period due to dryness or cold does not allow photosynthesis. Such belowground organs are intermingled with the soil and with the belowground parts of neighbouring plants and can occur down to soil depths >1 m and over areas larger than those covered by aboveground plant parts (Kutschera and Lichtenegger 1982, 1992; Maurin et al. 2014). Therefore, it is often very difficult, or simply time too consuming, to separate all the storage organs of a plant individual from the soil and determine the TNC pool. Thus, whereas dozens of studies have recorded TNC concentration in storage organs after disturbance events (see Janeček *et al*. 2011 and references therein), assessments of pools in this situation are very rare, especially in field conditions.

Total non-structural carbohydrate concentration is not always a reliable indicator of TNC pools because total amount of storage organ biomass could increase while TNC concentration decreases and vice versa. Thus, there are documented examples in which these two measures do not vary together. For example, field studies found that although TNC concentrations in shoot bases of meadow grasses are higher in mown than in unmown plots at the end of the growing season, the TNC pools show the opposite trend in some of the few species considered (Klimeš and Klimešová 2002; Bartoš *et al*. 2011).

Therefore, in the present study, in assessing long-term effects of disturbance cessation on seasonal storage, we examine the responses of both carbohydrate pools and concentrations. Additionally, we examine multiple plant species and use sites located in different habitats, in order to test the extent to which responses can be generalized across species and environments. Specifically, as our study system, we selected two temperate grassland sites that historically have been subjected to regular disturbance (mowing for hay), and examined responses of perennial plants that have compact storage organs that can be excavated from the soil. We manipulated disturbance so that portions of the meadows (one dry and one wet) were either subjected to cutting once a year or this management was abandoned. In both the mown and unmown plots, we analysed TNC in belowground storage organs in the middle and at the end of the first and third growing seasons after mowing had ceased in the unmown plots.

We used this system to test the following specific hypotheses: (1) storage will be greater in undisturbed than disturbed plants at the end of the first year after cessation of disturbance because of saved reserves usually needed for regeneration, but this difference will not increase further in the third year because high storage hinders competitive ability; (2) storage will increase over the course of the growing season in both disturbed and undisturbed plants, such that it will be higher at the end of it than during its peak and (3) changes in carbohydrate concentrations are accurate predictors of changes in pools.

## Methods

### Field experiment

The field experiment was established in 2005 in two meadows. These meadows differ in several characteristics, especially water availability and species composition. This approach allows us to better reveal both site-specific features and general patterns in carbohydrate economy. The dry meadow, known as Čertoryje, is located in the Bílé Karpaty Mts. [Czech Republic, 48°54′N, 17°25′E, 440 m above sea level (a.s.l.)]. It is species rich and is dominated by the grasses *Bromus erectus* and *Molinia arundinacea* and the sedge *Carex montana*. It has deep, calcium-rich soil. Its mean annual precipitation is 464 mm, and its mean annual temperature is 9.4 °C. For a more detailed description, see Klimeš (1995, 1999) and de Bello *et al*. (2012).

The wet meadow is known as Ohrazení and is located near the town of České Budějovice (Czech Republic, 48°57′N, 14°36′E, 500 m a.s.l.). It is dominated by the tall grass *Molinia caerulea* and has acidic soil. Its mean annual precipitation is 700 mm, and its mean annual temperature is 7–8 °C. For more information, see Lepš (1999) and de Bello *et al*. (2012).

For many years, both of the meadows had been mown in June of each year, up through 2004, the year prior to the beginning of the experiment. At the beginning of the experiment, we established experimental blocks (six at Čertoryje and five at Ohrazení), each divided into eight 9-m^2^ subplots. Four subplots from each block were mown ∼5 cm above the soil every year in June (i.e. during the peak of the growing season, when surrounding meadows are traditionally mown), and four subplots were left unmanaged. In June (before mowing was undertaken) and October of 2006 and 2008, i.e. 1 and 3 years after the disturbance regime was changed in the unmown subplots, we collected plants from one mown and one unmown subplots of each block.

Although both meadows are very species rich, we were not able to equally cover all the plant families or types of stored carbohydrates or storage organs occurring in these meadows (Janeček *et al*. 2011). Instead, we focussed on nine plant species selected based both on their sufficient abundance at the start of the experiment and on the possibility of completely digging up their main storage organs. This included six species in the dry meadow (*Lathyrus niger* and *Trifolium montanum*, both from Fabaceae; *Geranium sanguineum* from Geraniaceae; *Salvia pratensis* from Lamiaceae; *Clematis recta* from Ranunculaceae and *Filipendula vulgaris* from Rosaceae) and three in the wet meadow (*Angelica sylvestris* and *Selinum carvifolia* from Apiaceae and *Potentilla erecta* from Rosaceae). We were not able to wholly excavate any species from the well-represented family Poaceae or Cypearaceae.

At each harvest time, one specimen of each plant species from each harvested subplot was randomly selected and the whole plant, including belowground storage organs carefully excavated. The following storage organs were harvested in individual species: (i) thick skeletal roots with shoot bases (*L. niger*, *T. montanum*, *S. pratensis* and *A. sylvestris*); (ii) thick skeletal roots and rhizomes (*G. sanguineum*, *C. recta* and *S. carvifolia*); (iii) thick roots with root tubers and rhizomes (*F. vulgaris*) and (iv) rhizomes (*P. erecta*). The washed storage organs were frozen in liquid nitrogen immediately after harvesting, transported to the laboratory, lyophilized and ground up. Aboveground plant materials were placed in plastic bags and a cooler and transported to the laboratory, where they were oven-dried at 80 °C for 24 h and then weighed.

### Carbohydrate analyses

For the target species, the main storage carbohydrate is starch, except in the case of *S. pratensis*, which accumulates raffinose-family oligosaccharides (RFOs). For more details about the composition of carbohydrates in storage organs of the target species, see Janeček *et al*. (2011). In the present study, the starch content was determined using the total starch assay procedure developed by Megazyme International (see www.megazyme.com). In this procedure, the ethanol-soluble carbohydrates (glucose, fructose and sucrose) were first extracted from 100 mg of homogenized, milled dry biomass in glass centrifuge tubes. Following the manufacturer's recommendations, we used 5 mL of 80 % aqueous, boiling ethanol. During the 10 min of extraction, samples were mixed three times on a vortex stirrer. After extraction, tubes were centrifuged 10 min at 1800*g* and the supernatant was transferred to a glass bottle. This extraction was repeated three times. The aqueous ethanol was then evaporated and the ethanol-soluble sugars transferred to distilled water. Starch remaining in the undissolved pellet after extraction was enzymatically reduced to glucose by thermostable α-amylase and amyloglucosidase. The glucose was subsequently determined calorimetrically with the GOPOD reagent containing glucose oxidase, peroxidase and 4-aminoantipyrine. The ethanol-soluble carbohydrates, glucose, fructose and sucrose were analysed via high-performance anion exchange chromatography with pulsed amperometric detection using a Dionex ICS-3000 system and CarboPac PA1 analytical column (see Hardy and Townsend 1988; Corradini *et al*. 2012). When analysing ethanol-soluble carbohydrates, we used as eluent 200 mM NaOH with a flow rate of 1 mL min^−1^ for 15 min. The potentials and durations used for detection were *E*_1_ = 0.1 V (*t*_1_ = 410 ms); *E*_2_ = −2 V (*t*_2_ = 20 ms); *E*_3_ = 0.6 V (*t*_3_ = 10 ms) and *E*_4_ = −0.1 V (*t*_4_ = 60 ms). We used external standards of individual carbohydrates of concentration 0.1 mg mL^−1^ injected in three different volumes (5, 10 and 20 μL). For analyses of RFOs, which are accumulated in storage organs of *S. pratensis*, we used 16 mM NaOH, flow rate 1 mL min^−1^, for 30 min to carefully separate galactose from glucose. The potentials and durations for detection were the same as for ethanol-soluble carbohydrates. Raffinose-family oligosaccharide amounts were calculated as the difference between ethanol-soluble carbohydrates (galactose, glucose, fructose and sucrose) before and after addition of α-galactosidase (*Aspergillus niger*, Megazyme) to the extract. The concentration of TNC was calculated as the sum of the proportions of analysed carbohydrates in dry biomass (i.e. mg g^−1^). The TNC pool was calculated as the mass of the storage organs (in grams) multiplied by the concentration.

### Statistical analyses

First, for each meadow, we constructed general mixed-effect permutation analysis of variance models for individual parameters (i.e. aboveground biomass, belowground biomass, total biomass, TNC concentration and TNC pool). We considered the following as fixed factors: year (2006 versus 2008), season (June versus October), treatment (mown versus unmown) and species. Block was treated as a random factor. Because there were often significant interactions between species identity and other factors, we then examined the behaviour of individual species in greater detail. In performing separate analyses for each individual species, we considered three fixed factors (year, season and treatment) and one random factor (block). We tested all individual factors as well as their interactions. Data on aboveground biomass, belowground biomass, total biomass, TNC concentration and TNC pool were log-transformed to improve homoscedasticity and decrease the effect of extreme values, and also because the difference in species' proportional changes is biologically more relevant when species of different sizes are considered. These analyses were performed in PERMANOVA+ for PRIMER (Anderson *et al*. 2008).

## Results

### Plant biomass

**Figure 2.**
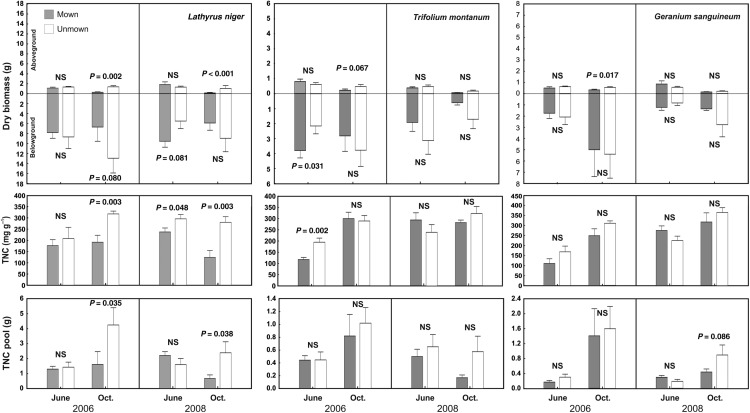
Effect of mowing on biomass and carbohydrate reserves of *Lathyrus niger*, *Trifolium montanum* and *Geranium sanguineum* growing in dry meadow. *Lathyrus niger* and *Trifolium montanum* have thick skeletal roots, whereas *Geranium sanguineum* has thick skeletal roots and rhizomes as storage organs. Means (bars) and SE (whiskers) are shown. NS, non-significant (contrasts with *P* < 0.10).

**Figure 3.**
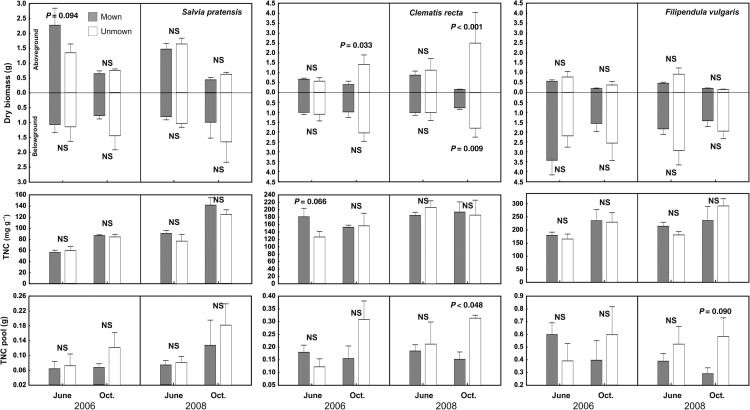
Effect of mowing on biomass and carbohydrate reserves of *Salvia pratensis*, *Clematis recta* and *Filipendula vulgaris* growing in dry meadow. *Salvia pratensis* has thick skeletal root, *Clematis recta* has thick skeletal roots and rhizomes and *Filipendula vulgaris* has thick roots with root tubers and rhizomes as storage organs. Means (bars) and SE (whiskers) are shown. NS, non-significant (contrasts with *P* < 0.10).

**Figure 4.**
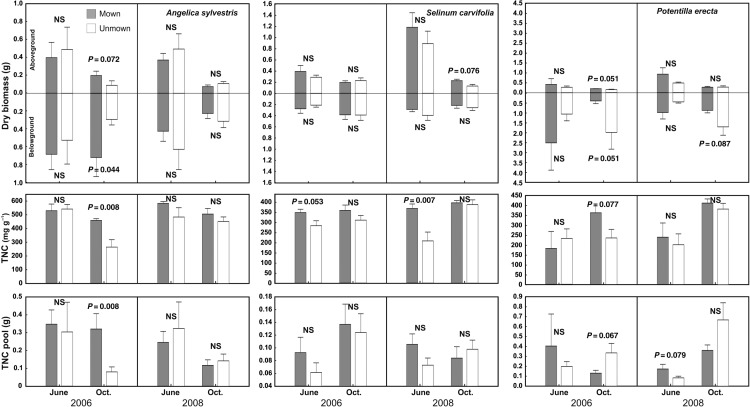
Effects of mowing on biomass and carbohydrate reserves of *Angelica sylvestris*, *Selinum carvifolia* and *Potentilla erecta* growing in wet meadow. *Angelica sylvestris* has thick skeletal roots, *Selinum carvifolia* has thick skeletal roots and rhizome and *Potentilla erecta* has rhizome as storage organs. Means (bars) and SE (whiskers) are shown. NS, non-significant (contrasts with *P* < 0.10).

Aboveground biomass was affected by all studied factors in both localities except for treatment effect in wet meadow, and often the effects were species specific (interaction of species with other factors in Tables 1 and 2; Fig. 1). At the species level, the strongest effect was found for ‘season’, while ‘treatment’ and ‘year’ were less important (Table 3; Figs 2–4).

**Table 1.** Dry meadow. Effect of treatment (mowing), year, season (June, October) and site on biomass and TNC storage properties of meadow plants (PERMANOVA). Block is a random factor; year, season, treatment and species are fixed factors. *F*-values are shown for significant (or marginally significant) results; ^†^0.05 < *P* < 0.1; *0.01 < *P* < 0.05; **P < 0.01; n.s., non-significant *P* ≥ 0.1; df, numerator/denominator degree of freedom; all dependent variables were log-transformed. Significant *F*-values (i.e. *P* < 0.05) are in bold.

	df	Aboveground biomass	Belowground biomass	Total biomass	TNC concentration	TNC pool
Year (Ye)	1/230	18.43**	10.67**	11.39**	43.05**	n.s.
Season (Se)	1/230	175.58**	n.s.	5.18*	45.59**	9.72**
Treatment (Tr)	1/230	44.51**	6.94**	11.34**	5.51*	12.55**
Block	5/230	n.s.	n.s.	n.s.	n.s.	n.s.
Species (Sp)	5/230	34.37**	40.44**	31.40**	67.08**	72.74**
Ye × Se	1/230	24.10**	n.s.	5.46*	12.07**	9.35**
Ye × Tr	1/230	n.s.	n.s.	n.s.	n.s.	n.s.
Ye × Sp	5/230	3.45**	3.20**	3.56**	2.64*	2.29*
Se × Tr	1/230	44.54**	14.25**	20.81**	5.36*	21.44**
Se × Sp	5/230	4.98**	3.77**	3.98**	6.05**	5.80**
Tr × Sp	5/230	5.50**	n.s.	n.s.	4.50**	n.s.
Ye × Se × Tr	1/230	n.s.	n.s.	n.s.	3.32^†^	n.s.
Ye × Se × Sp	5/230	n.s.	n.s.	n.s.	3.19**	n.s.
Ye × Tr × Sp	5/230	n.s.	n.s.	n.s.	2.36*	n.s.
Se × Tr × Sp	5/230	8.04**	n.s.	n.s.	1.87^†^	n.s.
Ye × Se × Tr × Sp	5/230	n.s.	n.s.	n.s.	n.s.	n.s.

**Table 2.** Wet meadow. Effect of treatment (mowing), year, season (June, October) and site on biomass and TNC storage properties of meadow plants (PERMANOVA). Block is a random factor; year, season, treatment and species are fixed factors. *F*-values are shown for significant (or marginally significant) results; ^†^0.05 < *P* < 0.1; *0.01 < *P* < 0.05; **P < 0.01; n.s., non-significant *P* ≥ 0.1; df, numerator/denominator degree of freedom; all dependent variables were log-transformed. Significant *F*-values (i.e. *P* < 0.05) are in bold.

	df	Aboveground biomass	Belowground biomass	Total biomass	TNC concentration	TNC pool
Year (Ye)	1/95	12.91**	n.s.	n.s.	3.39^†^	n.s.
Season (Se)	1/95	65.71**	n.s.	19.01**	7.43**	n.s.
Treatment (Tr)	1/95	n.s.	n.s.	n.s.	**3.57^†^**	n.s.
Block	4/95	2.84*	n.s.	n.s.	n.s.	n.s.
Species (Sp)	2/95	8.43**	27.48**	18.20**	23.56**	14.24**
Ye × Se	1/95	9.72**	n.s.	n.s.	3.44^†^	n.s.
Ye × Tr	1/95	n.s.	n.s.	n.s.	n.s.	n.s.
Ye × Sp	2/95	n.s.	n.s.	n.s.	n.s.	n.s.
Se × Tr	1/95	n.s.	3.22^†^	n.s.	n.s.	n.s.
Se × Sp	2/95	3.32*	n.s.	n.s.	12.95**	9.64**
Tr × Sp	2/95	n.s.	n.s.	n.s.	n.s.	n.s.
Ye × Se × Tr	1/95	n.s.	n.s.	n.s.	6.69**	n.s.
Ye × Se × Sp	2/95	n.s.	4.76*	4.02*	n.s.	3.66*
Ye × Tr × Sp	2/95	n.s.	n.s.	n.s.	n.s.	3.40*
Se × Tr × Sp	2/95	n.s.	4.12*	3.49*	2.97^†^	n.s.
Ye × Se × Tr × Sp	2/95	n.s.	n.s.	n.s.	n.s.	n.s.

**Table 3.** Effect of treatment (mowing), year and season (June, October) on biomass of individual plant species (PERMANOVA). Block identification is a random factor; year, season and treatment are fixed factors. *F*-values are shown for significant (or marginally significant) results; ^†^0.05 < *P* < 0.1; *0.01 < *P* < 0.05; **P < 0.01; n.s., non-significant (*P* ≥ 0.1); dfn, numerator degree of freedom; dfd, denominator degree of freedom. Significant *F*-values (i.e. *P* < 0.05) are in bold.

	dfn	Dry meadow						dfn	Wet meadow		
		*Lathyrus niger*	*Trifolium montanum*	*Geranium sanguineum*	*Salvia pratensis*	*Clematis recta*	*Filipendula vulgaris*		*Angelica sylvestris*	*Selinum carvifolia*	*Potentilla erecta*
dfd		35	37	35	35	27	36		28	32	27
Aboveground biomass (log)											
Year (Ye)	1	2.71^†^	16.80**	6.13*	n.s.	n.s.	n.s.	1	n.s.	8.94*	10.53**
Season (Se)	1	56.67**	31.92**	26.45**	74.28**	n.s.	46.34**	1	20.91**	35.58**	7.18*
Treatment (Tr)	1	35.77**	3.49^†^	n.s.	n.s.	12.24**	n.s.	1	n.s.	n.s.	n.s.
Block	5	n.s.	n.s.	n.s.	n.s.	n.s.	n.s.	4	n.s.	3.27*	n.s.
Ye × Se	1	6.55*	5.13*	12.54**	n.s.	n.s.	n.s.	1	n.s.	11.87**	n.s.
Ye × Tr	1	n.s.	n.s.	n.s.	3.70^†^	3.58^†^	n.s.	1	n.s.	n.s.	n.s.
Se × Tr	1	36.63*	**3.64^†^**	n.s.	4.82*	19.87**	n.s.	1	n.s.	n.s.	n.s.
Ye × Se × Tr	1	n.s	n.s.	n.s.	n.s.	n.s.	n.s.	1	n.s.	n.s.	n.s.
Belowground biomass (log)											
Year (Ye)	1	n.s.	12.88*	9.79**	n.s.	n.s.	n.s.	1	n.s.	n.s.	n.s.
Season (Se)	1	n.s.	n.s.	11.55**	n.s.	n.s.	n.s.	1	3.10^†^	n.s.	n.s.
Treatment (Tr)	1	n.s.	n.s.	n.s.	4.60*	n.s.	n.s.	1	n.s.	n.s.	n.s.
Block	5	n.s.	n.s.	n.s.	n.s.	n.s.	n.s.	4	n.s.	n.s.	n.s.
Ye × Se	1	n.s.	4.03**	n.s.	n.s.	n.s.	n.s.	1	n.s.	8.03**	3.64^†^
Ye × Tr	1	n.s.	n.s.	n.s.	n.s.	n.s.	n.s.	1	3.87^†^	n.s.	n.s.
Se × Tr	1	5.55*	n.s.	n.s.	n.s.	5.07*	n.s.	1	n.s.	n.s.	8.89**
Ye × Se × Tr	1	n.s.	n.s.	n.s.	n.s.	n.s.	n.s.	1	n.s.	n.s.	n.s.
Total biomass (log)											
Year (Ye)		n.s.	14.34**	8.4**	n.s.	n.s.	n.s.	1	n.s.	5.62*	n.s.
Season (Se)	1	n.s.	4.31^†^	4.47^†^	9.55**	n.s.	5.70*	1	8.91**	15.02**	n.s.
Treatment (Tr)	1	n.s.	n.s.	n.s.	n.s.	5.84*	n.s.	1	n.s.	n.s.	n.s.
Block	5	n.s.	n.s.	n.s.	n.s.	n.s.	n.s.	4	n.s.	3.29*	n.s.
Ye × Se	1	n.s.	5.13*	n.s.	n.s.	n.s.	n.s.	1	n.s.	16.54**	n.s.
Ye × Tr	1	n.s.	n.s.	n.s.	n.s.	n.s.	n.s.	1	3.18^†^	n.s.	n.s.
Se × Tr	1	6.59*	n.s.	n.s.	3.63^†^	11.39**	n.s.	1	n.s.	n.s.	8.21**
Ye × Se × Tr	1	n.s.	n.s.	n.s.	n.s.	n.s.	n.s.	1	n.s.	n.s.	n.s.

Belowground biomass of storage organs in dry meadow differed among years, treatments and species, but there was no main effect of season. However, season did affect belowground biomass differently in relation to treatment, as biomass decreased in mown plots and increased in unmown plots from June to October (Fig. 1). In the wet meadow, belowground biomass was affected mainly by species identity (Table 2). At the species level, the reaction of belowground biomass to studied factors and their interactions was not consistent; for example, the effect of season on storage organ biomass was modulated by treatment only in *L. niger*, *C. recta* and *P. erecta* (Table 3; Figs 2–4). In *G. sanguineum*, however, the main factors affecting the mass of storage organs were ‘year’ and ‘season’ (Table 3; Fig. 2).

### Carbohydrate reserves

**Figure 5.**
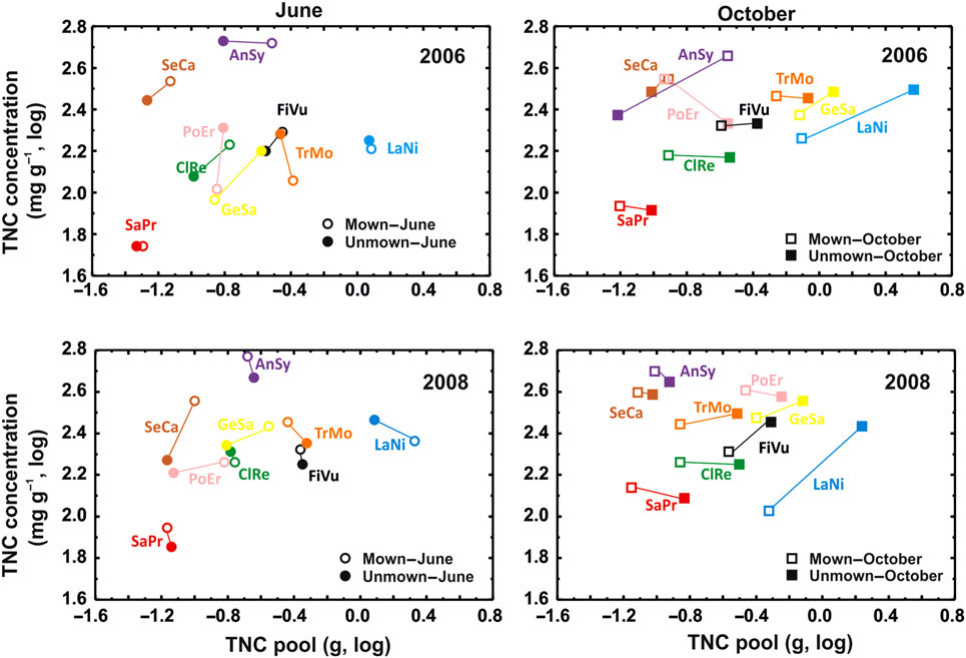
Changes for individual species in TNC concentrations and TNC pools in diferent treatments. LaNi, *Lathyrus niger*; TrMo, *Trifolium montanum*; GeSa, *Geranium sanguineum*; SaPr, *Salvia pratensis*; ClRe, *Clematis recta*; FiVu, *Filipendula vulgaris*; AnSy, *Angelica sylvestris*; SeCa, *Selinum carvifolia*; PoEr, *Potentilla erecta*.

**Figure 6.**
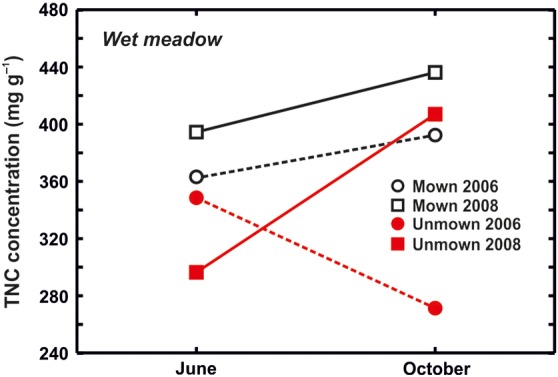
Wet meadow. Effect of treatments on seasonal changes in TNC concentrations in 2006 and 2008.

In both meadows, TNC concentrations were affected by all studied factors and also by their interactions. In the dry meadow, the TNC concentrations were generally higher at the end of season in both treatments, but the increase was higher in unmown plots (Figs 1 and 5). In the wet meadow, seasonal changes were affected by the treatment, but differently in individual years. During the 2006 growing season, TNC concentrations increased in unmown plots, whereas in 2008, they decreased (Table 2; Fig. 6). Changes in TNC concentrations were species specific at both sites. In some species (e.g. *G. sanguineum* or *S. pratensis*), TNC concentrations increased in October of both years (Table 4; Figs 3 and 4). In *T. montanum*, in contrast, the relative positions of June and October concentrations were different in each year (Table 4; Fig. 2). There was a decrease in *L. niger* in TNC concentration due to abandonment, but no treatment effect for *F. vulgaris* (Table 4; Figs 2 and 3). For some species, the effect of treatment differed between years.

**Table 4.** Effect of treatment (mowing), year and season (June, October) on TNC of individual plant species (PERMANOVA). Block identification is a random factor; year, season and treatment are fixed factors. *F*-values are shown for significant (or marginally significant) results; ^†^0.05 < *P* < 0.1; *0.01 < *P* < 0.05; **P < 0.01; n.s., non-significant (*P* ≥ 0.1); dfn, numerator degree of freedom; dfd, denominator degree of freedom. Significant *F*-values (i.e. *P* < 0.05) are in bold.

	dfn	Dry meadow						dfn	Wet meadow		
		*Lathyrus niger*	*Trifolium montanum*	*Geranium sanguineum*	*Salvia pratensis*	*Clematis recta*	*Filipendula vulgaris*		*Angelica sylvestris*	*Selinum carvifolia*	*Potentilla erecta*
dfd		35	37	35	35	27	36		28	32	27
TNC concentration (log)											
Year (Ye)	1	n.s.	23.44**	15.08**	25.39**	4.46*	n.s.	1	3.2^†^	n.s.	n.s.
Season (Se)	1	n.s.	29.1**	28.29**	33.60**	n.s.	3.63^†^	1	7.68**	4.50*	11.17**
Treatment (Tr)	1	15.60**	n.s.	3.11^†^	n.s.	n.s.	n.s.	1	6.15*	6.05*	n.s.
Block	5	n.s.	2.4^†^	n.s.	n.s.	n.s.	n.s.	4	n.s.	n.s.	n.s.
Ye × Se	1	9.23**	6.38*	5.24*	n.s.	n.s.	n.s.	1	3.01^†^	7.60**	n.s.
Ye × Tr	1	n.s.	2.98^†^	3.55^†^	n.s.	n.s.	n.s.	1	n.s.	n.s.	n.s.
Se × Tr	1	7.15*	n.s.	n.s.	n.s.	n.s.	n.s.	1	n.s.	7.33*	n.s.
Ye × Se × Tr	1	n.s.	12.89**	n.s.	n.s.	n.s.	n.s.	1	4.57*	n.s.	n.s.
TNC pool (log)											
Year (Ye)	1	n.s.	5.38*	n.s.	n.s.	3.47^†^	n.s.	1	n.s.	n.s.	n.s.
Season (Se)	1	n.s.	n.s.	32.46**	4.23^†^	3.39^†^	n.s.	1	6.77*	n.s.	11.06**
Treatment (Tr)	1	5.73*	n.s.	n.s.	n.s.	n.s.	n.s.	1	3.28^†^	n.s.	n.s.
Block	5	n.s.	n.s.	n.s.	n.s.	2.51^†^	n.s.	4	n.s.	n.s.	n.s.
Ye × Se	1	2.70^†^	7.14**	n.s.	n.s.	n.s.	n.s.	1	n.s.	n.s.	5.34*
Ye × Tr	1	n.s.	n.s.	n.s.	n.s.	n.s.	n.s.	1	4.26^†^	n.s.	n.s.
Se × Tr	1	13.43**	n.s.	n.s.	n.s.	7.17*	n.s.	1	n.s.	n.s.	4.67*
Ye × Se × Tr	1	n.s.	n.s.	n.s.	n.s.	n.s.	n.s.	1	n.s.	n.s.	n.s.

Season and treatment had both overall and species-specific (i.e. in interaction with species) effects on TNC pools in the dry meadow, but only species-specific effects (i.e. in interaction with the species factor) were observed in the wet meadow (Tables 1 and 2). In the dry meadow, the TNC pool would grow during the season in unmown plots but remain the same in mown plots (Fig. 1). For some species, their patterns of significant results for TNC pools and concentrations were similar to each other (Table 4, Figs 1–3), although the TNC pools in *S. pratensis*, *F. vulgaris* and *S. carvifolia* showed no relationship to year, season, disturbance regime or the interaction of these factors.

When we examined the positions of species in trait space determined by TNC concentrations and TNC pools (Fig. 5), we found that (i) overall, the positions of species relative to other species did not shift much during the 3 years of the study; (ii) for June, the relationship between pool and concentration differed between years and species, whereas for October, species tended to have the same TNC concentration in mown as unmown plots, although TNC pools were higher for unmown plants than mown plants and (iii) for unmown plants, TNC pools increased for most plants by the end of the growing season the year following mowing abandonment, but by the next June measurement (i.e. 3 years after the beginning of the experimental treatment), they shrank to levels similar to those in mowed plants.

## Discussion

### Effect of disturbance exclusion

Our expectation that storage would increase after abandonment was not well supported in our study as plants in unmown and mown plots had similar carbohydrate storage in June both 1 and 3 years after the disturbance regime was changed. However, plants in unmown plots had higher reserves than plants from mown plots in both Octobers, at growing season end (see also ‘Effect of season’) and showed greater seasonal fluctuations than the disturbed plants. The lack of general increase in carbohydrates storage after disturbance cessation could have been caused by several mechanisms: (i) trade-off between storage and growth (and hence competitive ability) caused plants with large storage organs to die during the study; (ii) higher competitive milieu after mowing cessation caused plants to change carbohydrate allocation strategy and invest more in aboveground biomass and (iii) increased competition caused less carbohydrates to be available for storing (see also ‘Effect of season’).

A requirement for all of these mechanisms is increasing competition for light after meadow abandonment. This, however, is doubtful, as another study from the same system indicated that, in the studied time span, plants were experiencing competition only in the more productive, wet meadow and not in the dry meadow (Bartušková *et al*. 2015). In particular, that study found increasing competition in unmown plots in the wet meadow was accompanied with changes in plant aboveground biomass allocations such that the proportion of allocation towards supportive organs (stems and petioles) increased relative to carbon assimilating leaves. However, the storage response detected in the present study was not consistent with this pattern, probably due to the smaller number of examined species. In the less productive, dry meadow, biomass allocation of resident plants was not affected consistently as light limitation after cessation of mowing in species-rich unproductive meadows does not play an important role (Eek and Zobel 2001; Bartušková *et al*. 2015). Moreover, not only is the dry meadow limited by water availability, but changes in species composition after abandonment are slow (Klimeš *et al*. 2013). There is also the possibility that unmown plots in our experiment were more greatly affected by herbivory when all surrounding meadows were mown and did not have any foliage available to herbivores. However, according to our observations, deer like to graze preferentially on generative structures in meadow plants, so that their main effect could have been on reproductive biomass.

### Effect of season

In accord with previous studies, we expected that storage would increase from June to October, i.e. from the peak of the growing season to its end, at which time plants would be preparing themselves for a rather long (4–5 months) winter, and accumulate carbohydrates for respiration and spring regrowth (Janeček *et al*. 2011). Our results supported this hypothesis, as concentrations and pools of carbohydrates mostly increased as expected. In the dry meadow, in both treatments and both years, TNC concentrations increased in October in comparison with June. Increased TNC concentrations in mown plots caused TNC pools to remain the same over the growing season despite reduction of belowground biomass. In contrast, in unmown plots, increased TNC concentrations, together with increased biomass of belowground organs, caused substantial increases in the TNC pools. Plants in abandoned plots, due to their production of new belowground organs (rhizome increments and roots), therefore entered the winter with larger pools of assimilates than plants from mown plots. The difference, however, vanished by the measurement done in the June, 3 years after the beginning of the experimental treatment, when plants from both treatments had concentrations as well as pools of carbohydrates at similar levels.

The occurrence of the same levels of reserves in mown and unmown plots in June indicates a more pronounced decrease of reserves in unmown plots during winter and spring. A similar pattern was observed for *Festuca paniculata* by Baptist *et al*. (2013) who explained these observations by investment into establishment of new tillers in unmown plots during the winter. This explanation is not applicable to the species we studied, because we had chosen species with compact belowground organs and therefore with very limited or no clonality; thus, we should consider other mechanisms. For example, plants in unmowed plots could use stored carbohydrates to enable shoot penetration through the layer of accumulated old biomass (litter), which is removed from mown meadows as hay. Such litter is known to have important effects on plant growth and plant community structure by affecting the microclimate for seedling establishment as well as seasonal regrowth of early and very small species (Facelli and Pickett 1991; Janeček and Lepš 2005). In our system, it is apparent that in the absence of mowing, plants invested more carbohydrates into regrowth the following spring, even using storage that would otherwise have been used for summer regrowth after mowing.

As described in the section ‘Effect of disturbance exclusion’, however, an increasing competitive milieu due either to litter accumulation or more vigorous growth of plants not subjected to regular management is improbable in a less productive, dry meadow. Therefore, we must find an explanation for the larger seasonal variation in the carbohydrate pools in unmown plots.

As possible explanations for the large seasonal variation in carbohydrate pools in unmown plots in dry meadows, we suggest higher investment in generative reproduction (Iwasa and Kubo 1997) or higher turnover of tissue-forming storage organs, i.e. formation of new increments of stems/rhizomes and storage roots to replace old ones. In a preceding study of biomass allocation to aboveground plant organs, we did not record an increase in generative plant parts on unmown plots (Bartušková et al. 2015). This, however, could have been due partly to preferential grazing of wild animals (deer) on plant inflorescences. Larger turnover of belowground biomass could occur due to greater respiration during the winter because of increased volume of storage organs. However, there is another benefit, beyond storage, that can arise from greater belowground investment (not just in storage organs), and this benefit is increased mobility, sometimes just at the scale of centimetres. Mobility has been predicted to reduce competition in meadows (van der Maarel and Sykes 1993).

### Species-specific effects

The species specificity of the effect of mowing on TNC storage is in agreement with previous studies that have assessed multiple grassland species (Trlica *et al*. 1977; Klimeš and Klimešová 2002). This species specificity might be caused by the fact that different species are mown at different phenological stages (Menke and Trlica 1981; Martínková *et al*. 2002). Alternatively, it might be caused by differences in the carbohydrate economy of individual plant functional groups (Janeček *et al*. 2011). It is probable that carbohydrate storage plays an important role in species coexistence in species-rich meadows, a subject that deserves further study. In any case, the differences in species responses suggest that we should not overgeneralize based on single-species studies.

### Storage traits: TNC concentration versus TNC pool

Our study demonstrates that TNC concentrations and TNC pools reflect different aspects of carbohydrate storage and in this respect are in accord with results of other studies done in the same ecosystem so far (Klimeš and Klimešová 2002; Bartoš *et al*. 2011; Janeček and Klimešová 2014). The carbohydrate storage of herbs from species-rich, dry, regularly mown meadows is sufficient for winter survival and spring regrowth although it is not sufficient for other functions. Thus, for example, in potentially clonal plants (Klimeš 1999), clonal growth is reduced, and their investments into flowering are low (Bartušková *et al*. 2015). Moreover, for clonal species, after disturbance is excluded, plants are capable of higher investment in belowground organs and their clonal mobility increases (Baptist *et al*. 2013) along with allocation to generative reproduction.

## Conclusions

Plants in meadows in which disturbance has recently ceased are able to store larger TNC pools than plants in mown meadows. These large TNC pools, however, are depleted during winter and/or spring so that summer pools and concentrations do not differ between plants from differently managed plots. It is clear, moreover, that although TNC concentrations at first reflect the carbohydrate mobilization needed for resprouting in response to plant damage and then the refilling of reserves thereby expended, the pools are affected by the growth of storage organs, which can occur seasonally in accordance with the plant's phenology. Although TNC concentrations and TNC pools reflect different aspects of plant carbohydrate storage, TNC concentration, as the more easily measurable trait, might sufficiently describe short-term effects of disturbance.

## Sources of Funding

This project was supported by the Grant Agency of the Czech Republic (GA 526/09/0963, P505/12/1296), long-term research development project RVO 67985939 and the Centre of Excellence (14-36079G), PLADIAS.

## Contributions by the Authors

Š.J., A.B., M.B., J.A., F.d.B., J.D., V.Lat., V.Lan. and J.K. performed the experiment. Š.J. and J.L. analysed the data. Š.J. and J.K. prepared the manuscript. All authors read and approved the final manuscript.

## Conflict of Interest Statement

None declared.
